# A whole-plant chamber system for parallel gas exchange measurements of Arabidopsis and other herbaceous species

**DOI:** 10.1186/s13007-015-0089-z

**Published:** 2015-10-16

**Authors:** Katharina Kölling, Gavin M. George, Roland Künzli, Patrick Flütsch, Samuel C. Zeeman

**Affiliations:** Department of Biology, Institute of Agricultural Sciences, ETH Zurich, Universitätstrasse 2, 8092 Zurich, Switzerland; DMP Ltd, Allmendstrasse 9, 8320 Fehraltorf, Switzerland

**Keywords:** Photosynthesis, Carbon assimilation, Transpiration rate, Respiration, Gas exchange

## Abstract

**Background:**

Photosynthetic assimilation of carbon is a defining feature of the plant kingdom. The fixation of large amounts of carbon dioxide supports the synthesis of carbohydrates, which make up the bulk of plant biomass. Exact measurements of carbon assimilation rates are therefore crucial due to their impact on the plants metabolism, growth and reproductive success. Commercially available single-leaf cuvettes allow the detailed analysis of many photosynthetic parameters, including gas exchange, of a selected leaf area. However, these cuvettes can be difficult to use with small herbaceous plants such as *Arabidopsis thaliana* or plants having delicate or textured leaves. Furthermore, data from single leaves can be difficult to scale-up for a plant shoot with a complex architecture and tissues in different physiological states. Therefore, we constructed a versatile system—EGES-1—to simultaneously measure gas exchange in the whole shoots of multiple individual plants. Our system was designed to be able record data continuously over several days.

**Results:**

The EGES-1 system yielded comparable measurements for eight plants for up to 6 days in stable, physiologically realistic conditions. The chambers seals have negligible permeability to carbon dioxide and the system is designed so as to detect any bulk-flow air leaks. We show that the system can be used to monitor plant responses to changing environmental conditions, such as changes in illumination or stress treatments, and to compare plants with phenotypically severe mutations. By incorporating interchangeable lids, the system could be used to measure photosynthetic gas exchange in several genera such as *Arabidopsis, Nicotiana*, *Pisum*, *Lotus* and *Mesembryanthemum*.

**Conclusion:**

EGES-1 can be introduced into a variety of growth facilities and measure gas exchange in the shoots diverse plant species grown in different growth media. It is ideal for comparing photosynthetic carbon assimilation of wild-type and mutant plants and/or plants undergoing selected experimental treatments. The system can deliver valuable data for whole-plant growth studies and help understanding mutant phenotypes. Overall, the EGES-1 is complementary to the readily-available single leaf systems that focus more on the photosynthetic process in within the leaf lamina.

**Electronic supplementary material:**

The online version of this article (doi:10.1186/s13007-015-0089-z) contains supplementary material, which is available to authorized users.

## Background

Plants capture energy from the sun to perform photosynthesis, during which light energy is converted to chemical energy equivalents. These are primarily used to assimilate carbon dioxide to form carbohydrates, which are subsequently metabolized to form all the organic components of the plant. Due to its overall importance, accurate measurements of photosynthesis are needed.

Gas exchange measurements are a non-destructive, non-invasive technique to monitor photosynthetic carbon assimilation rate. Measurements are rapid and can be used in the laboratory as well as in the field to investigate photosynthetic rates under different environmental conditions [1, 2], but also to compare plant lines with altered photosynthetic capacity.

Most commercially available systems for measuring gas exchange are based on a leaf cuvette connected to an infrared gas analyzer. The cuvette is clamped over a single leaf and the gas exchange of a small area of the leaf blade (typically 2–10 cm^2^) is measured. This method is versatile and instrumentation for it has been refined over recent decades. Nevertheless, single-leaf measurements can be problematic for several reasons. First, carbon assimilation can vary over the leaf surface (e.g. due to differences in stomatal distribution; [3]). Measuring multiple replicates at different sites of the leaf is needed to get a useful average assimilation rate. Additionally, carbon assimilation rate may depend upon the age and developmental stage of the plant. In apple, it was shown that leaves increase their assimilation rate as they develop, reaching maximum rates at the final expansion [4]. Later, during senescence, a decrease in assimilation rate was observed [5]. In contrast, in Arabidopsis, comparable carbon assimilation rates were reported for leaves 4–12 in a mature rosette [6]. However, the oldest and youngest leaves were excluded, presumably due to leaf size restrictions. Thus, while single leaf carbon assimilation rates are ideal for integration with measurements of other photosynthetic parameters, carbon assimilation measurements on a whole-plant level are also valuable and can be more readily integrated with other research methods. Whole-plant analyses can reflect more closely natural growth conditions and can be applied to plants whose morphologies are not ideally suited to typical leaf cuvettes (i.e. small and/or delicate leaves, leaves with irregular surfaces, plants with photosynthetic stems etc.). Whole-plant measurements can be particularly valuable for Arabidopsis where there is an incredible wealth of genetic resources, yet where single leaf measurements can be challenging (e.g. on dwarf, slow-growing, or early flowering mutants). Incorporating carbon assimilation measurements into research with Arabidopsis or other model systems such as *Lotus japonicas* will help to improve our understanding of whole-plant metabolism, physiology and biomass accumulation.

Chamber systems to measure gas exchange in whole shoots have been developed for different species [7, 8]. For *Arabidopsis thaliana,* commercially available systems as well as custom-made systems have been established [9, 10]. All enable the assembly of a closed chamber around the shoot of the plant, allowing gas exchange measurements of the whole leaf area while excluding CO_2_ released from root respiration. Net carbon assimilation rates gained by these systems are variable between experiments. For whole-plant measurements of the Arabidopsis wild type Col-0, published values range from 3.5 to 9 µmol CO_2_ m^−2^ s^−1^. Some of this variation is likely due to differences in growth conditions, but also due to the technical limitations of the gas exchange systems, [6, 10, 11]. The most common limitation of such systems is that they only measure one plant at a time. As photosynthetic parameters are sensitive and can respond rapidly to fluctuations like temperature, relative humidity, light intensity and plant handling, it is essential to grow the plants under the same conditions to allow comparison of independent measurements. Increasing replication of measurements in single chamber systems inevitably extends the experimental window or decreases the acclimation time of the plants, both of which will introduce variability into an experiment. A multi-chamber system was described previously which uses whole-plant cuvettes but does not isolate root respiratory CO_2_ production from photosynthetic CO_2_ assimilation [11].

Here we describe the development of a new chamber and control system for the measurement of gas exchange incorporating the important features of previous systems with critical improvements. This flexible, multi-chamber system incorporates three differently-sized, interchangeable lids allowing for measurements of different herbaceous species. The chambers isolate shoots from roots preventing day-time respiratory CO_2_ contamination. Airflow rates are computer-controlled and can be decreased to measure either very small plants or night-time respiration accurately. Moreover, up to eight plants can be monitored simultaneously and continuously, enabling precise comparative measurements as well as long-term observations.

## Results and discussion

### Design of the ETH Zurich gas exchange system (EGES-1)

**Fig. 1:**
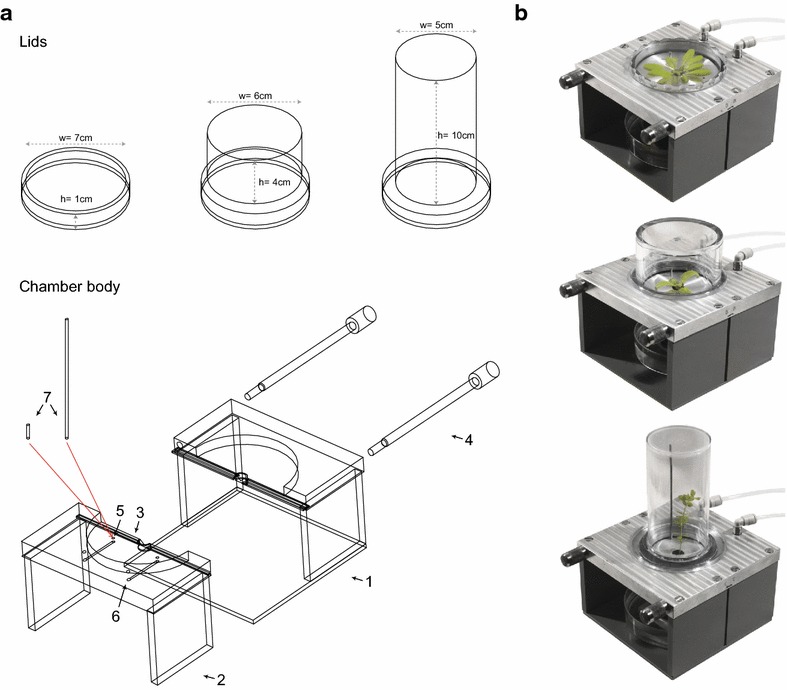
Arabidopsis gas exchange chamber. **a** Schematic overview of the three lid-types and the chamber. The chamber body contains two stabilizing units (*1* *+* *2*) and a rubber lip and foam gasket (*3*) to allow for an air-tight assembly. The two units are connected via screws (*4*) and closed by a lid. Air is flowing via an inlet (*5*) into the chamber and leaves it though an outlet (*6*). Depending on the lid-type an adapter tube (*7*) is added to the inlet. **b** Pictures of the gas exchange chamber filled with *Arabidopsis thaliana*, *Mesembryanthemum crystallinum* and *Lotus japonicus*

**Fig. 2:**
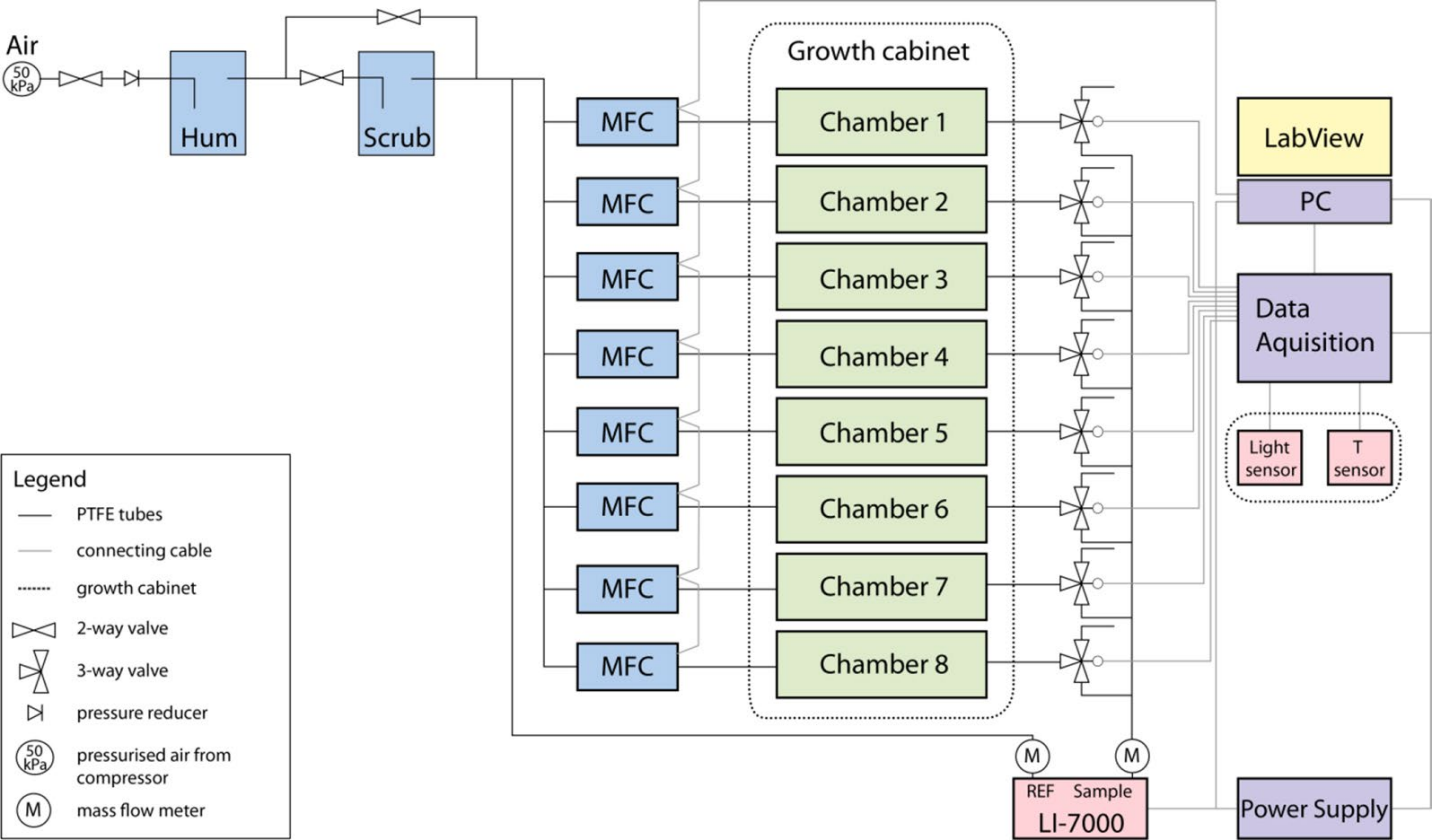
Schematic overview of the gas-exchange measuring unit (EGES-1). In *blue* the mechanical devices are depicted, in *green* the chambers containing the plants, in *red* the sensors, in *violet* all electrical components and in *yellow* the software

The system consists of eight whole-plant chambers with three sets of differently dimensioned lids (Fig. 1), a control unit, an infrared gas analyzer (IRGA) and a computer for airflow control and data acquisition. A schematic overview is shown in Fig. 2.

The whole-plant chambers were designed to allow for fast and simple assembly, without damaging the plants, and for easy adaptation to different plant species by using an appropriate screw-fitting lid (Fig. 1). One of the three differently sized lids is for flat plants like *Arabidopsis thaliana* with a rosette diameter of up to 7 cm (inner diameter 7 cm; height 1 cm; volume 38 ml). The second lid-type has a smaller diameter but is higher (inner diameter 6 cm; height 4 cm; volume 113 ml). It is suitable for more upright genera, such as young *Nicotiana sylvestris* and *Mesembryanthemum crystallinum* plants. The third lid-type is even narrower and taller (inner diameter 5 cm; height 10 cm; volume 196 ml) and suitable for trailing or tall plants like *Pisum sativum* (pea) and *Lotus japonicus*. To ensure an even airflow through the whole chamber, the incoming air is guided via an adapter tube (1 cm or 8 cm in length) to the top of the chamber, while the outgoing air exits through a hole on an adjacent quadrant on the base.

To assemble the chambers, the hypocotyl or stem of the plant is placed between two soft rubber-foam gaskets in the center of the two parts of the stabilizing base units, which are subsequently screwed together. The rubber foam forms a seal around the stem, which can be made air-tight, if necessary, with a small amount of soft paraffin. The two halves of the base unit seal together via a rubber lip that is compressed as the unit is assembled. The lids can then be screwed into place via a screw thread milled into a depression in the base unit to create the chamber. The lids also have a circular foam gasket to seal onto the base unit. The system can be used to measure plants in soil, growing in hydroponics, or other substrates. The minimal chamber volumes (38–196 ml) restrict the size of the plants that can be measured, but confer responsiveness to the measurements upon changing the environmental parameters. The relatively rapid flow rate relative to the chamber volume also ensures sufficient mixing of the air within the chamber.

The system requires a source of pressurized air. The compressed air supply in our institute is dry and enriched in CO_2_. The pressure is stabilized and reduced to slightly above atmospheric levels by a gas flow regulator. The air is then passed sequentially through a water-containing unit to increase the relative humidity and a soda lime unit to reduce CO_2_ content. Both units are set-up manually at the start of the experiment to provide the desired incoming air quality (380–400 ppm CO_2_ and 55–65 % RH in our experiments). Subsequently, the air is split into 9 channels. Eight channels supply the chambers. The ninth serves as a reference for the CO_2_ and H_2_O content in the incoming air. The airflow to the chambers is regulated by eight separate mass-flow controllers (MFC), allowing independent flow control for each chamber. The flow through each chamber is constant and the outflows are directed back to the control unit. Here, a gas-switching system sequentially channels returning air from each chamber through a mass flow meter (to ensure there are no significant air leaks in the chambers) and then to an IRGA. Thus, gas exchange of one chamber at a time is measured (Additional file 1: Figure S1). The outflowing air of all other chambers, when not being measured, is released, meaning that conditions for each plant are constant. By measuring the CO_2_ and H_2_O content of the reference air and of the air flowing out of the chambers it is possible to use the differences to calculate the net carbon assimilation/release and transpiration rates. All data are recorded every 1–2 s, displayed in real time, and collected and automatically saved in a user-determined time-averaged form by a customized LabView application. Via this application, the gas exchange system is easy and intuitive to operate (Additional file 1: Figure S2).

We decided upon a system design in which eight plants are measured together. This allows for a semi-continuous measurement of gas exchange (typically once every 48 min, with a realistic maximum of once per 16 min), yet also for a reasonably high throughput. Increasing the number of plants would increase the throughput, but lead to fewer measurements per time point or to less measurements over the day. We would not advise this, as the stability of the measurement from each chamber is a useful control for the air-tightness (see below), while excessively long intervals between measurements would not properly record changes in photosynthetic activity. A reduction in the number of plants measured is possible any time, as the chambers can be excluded easily from the measurements via the LabVIEW application. This offers the possibility to monitor fewer plants more precisely and follow rapid changes (e.g. after the application of stresses).

Our system can be easily introduced into most plant growth facilities, as the IRGA and control units are separated from the plant chambers by variable length tubing, enabling precise control of growth conditions. It can be used to monitor net photosynthetic carbon assimilation, dark respiration, and transpiration over the course of several days. This offers the possibility to investigate gas exchange during the diurnal cycle, during periods of plant development, in response to specific treatments, or in mutant/transgenic plants compared with wild-type controls. By measuring several plants in parallel, replicated measurements are acquired, minimizing errors that can occur during sequential gas exchange measurements on different individuals (e.g. due to handling, to differences in environmental conditions, time of day, etc.).

### Optimizing the setup of the gas exchange system

Test gas-exchange measurements with empty, properly closed cambers and an ambient air supply gave ΔCO_2_ and ΔH_2_O values constantly around zero, with negligible fluctuations over a 12-h measurement period. However, most of our measurements were conducted in conditions where there was a difference in CO_2_ between the outside room air (typically 500–550 ppm) and the quality-controlled airflow through the chamber (set to 380–400 ppm). To evaluate the potential impact of diffusion of CO_2_ through the seals of the chambers on our measurements, we first measured the difference between the supplied air reference and the ambient air, then measured the supplied air after flowing through a closed chamber. A diffusional leak into the chamber would register as a positive CO_2_ signal relative to the reference air. With a difference of 100 ppm between room air and supplied air, the diffusional leak was measured at less than 0.2 ppm (Additional file 1: Figure S3). Given that the typical ΔCO_2_ for a small plant measured in comparable conditions is 20 ppm or more, this is a potential error of 1 % or less. Even when air almost completely scrubbed of CO_2_ was pumped through the chamber (i.e. a difference of 500 ppm or more), the measured diffusional leak was still less than 2 ppm (not shown).

In additional to diffusional leaks, bulk air flow leaks may also occur through tiny gaps in the seals. Because our system works with a slight positive internal pressure, such leaks would be outward from the chamber and, if significant, would reduce the return airflow to the IRGA. This can be detected via the mass flow meter preceding the IRGA. To assess their impact on our measurements, bulk flow leaks were deliberately introduced into empty gas exchange chambers using hollow needles of different diameters inserted between the foam rubber gaskets and/or by loosening the lid seal. Repeating the above experiment using the mass flow meter to gauge the extent of the bulk flow leak revealed that the primary effect of a leak was on the speed of system equilibration (because of the lower flow rate from the chamber to the IRGA). There was also a slight increase in the measured CO_2_ to 0.4 ppm when the leak was approximately half of the supplied air and 0.8 ppm in the worst case, when the leak was equivalent to 90 % of the supplied air. However, in all experiments with plants, the mass flow meter preceding the IRGA was used at the outset to confirm that each chamber was essentially air tight. If any chamber was not air-tight it was dismantled and re-assembled. In longer test runs, we noticed that deliberate bulk flow leaks often caused the readings within a typical 360 s measurement period to be unstable, potentially serving as a useful indicator for a loss of chamber tightness during extended experiments (this did not occur, however). We emphasize that it is crucial, when designing and using similar systems, to evaluate and minimize diffusional and bulk air flow leaks in order to get robust results. It is also crucial to re-evaluate the extent of leaks when altering system parameters such as flow rates (e.g. reducing them for small plant samples) or supplied CO_2_ concentrations, which could increase the significance of both diffusional and bulk flow leaks.

When multiple chambers are being analyzed and the control unit switches from one to the next, air from the previous chamber is still in the tubing between the gas switching unit and the IRGA. This air needs displacing from the system before correct measurements for the new chamber can be obtained. The length of the required ‘dead time’ needs to be determined experimentally for any given flow rate. For this, the chambers were filled with plants, an airflow rate of 200 µmol s^−1^ was applied. Data were continuously recorded between chamber switches and 15-s averages calculated. A typical pattern seen after switching between two chambers showed that both ΔCO_2_ and ΔH_2_O values changed rapidly in the first 15 s, then began to stabilize (Additional file 1: Figure S4). After 90 s (the sixth measurement point), the measurements were quite stable. To account for this dead time, and facilitate subsequent data handling, a 90-s gap in data acquisition was introduced. The dead time can be reduced for higher flow rates, or extended for lower flow rates. Again, bulk air flow leaks from a chamber would affect the rate of air outflow to the IRGA and alter the dead time, also emphasizing the need to visually control for such leaks at the start of each experiment.

### Consistency of the measurements gained with the EGES-1

An example of consistent and stable measurements of all eight chambers filled with wild-type Arabidopsis plants (Col-0) for a complete 24 h period is shown in Additional file 1: Figure S5. Plants were 32 days old and an air flow of 200 µmol s^−1^ was used. Every 360 s the measured chamber was changed. After a 90-s dead time, average values were recorded every 30 s during the 270 s measurement period. Therefore, for each measurement period yielded 9 values.

All data points gained during the measurement are plotted against a time axis. As the measurements of one measurement period are stable, the points tend to overlay each other and appear as a single point. Generally, with all eight chambers we observed stable values with only slight changes during the light period. At the beginning of the day, after switching on the lights in the growth cabinet, there was a rapid increase of net carbon assimilation rate (A) as well as an increase in transpiration rate (E; see Additional file 1: Figure S5a, inset). Both values stabilize after 20 min and increase only slightly thereafter, with a maximum reached around 8 h into the light. Overall, this led to a variation in A over the light period of ±11 % and a variation in E of ±15 % in relation to the average. This pattern, with a maximum of A and E around 8 h into the light was observed in many of the subsequent measurements. So far, stable carbon assimilation rates over the day have been reported [11, 12], but these measurements were not analyzed in such detail for changes over the light and dark period.

During the day, the plants in the eight chambers had a mean net carbon assimilation rate of 7.45 µmol m^−2^ s^−1^ with a 4.6 % difference between the parallel measurements. The mean transpiration rate was 1.38 mmol m^−2^ s^−1^ with a variation of 8.7 %. The variation during the night was slightly higher with 15 % for respiration rate and 10.5 % for transpiration rate. This increased variation was in part due to the lower ΔCO_2_ and ΔH_2_O values obtained at night, compared to the day. In our system, sensitivity can again be recovered by reducing the airflow should fine measurements of respiration be needed. The net carbon assimilation rate gained from these measurements is in the range of previously published rates for Arabidopsis. In the literature, measurements for whole Arabidopsis plants grown under a 12 h light/12 h dark cycle range between 5 and 10 µmol m^−2^ s^−1^ [2, 9, 13]. The standard deviation of 0.34 is relatively low compared to the value of ~0.6 observed for the gas exchange systems used by Sun et al. [2] and Tocquin et al. [10]. Measurements with commercially available systems on single leaves also show similar or even higher variation between plants (SE ~0.2–1.5, n = 5; [6]). This shows that the EGES-1 delivers reliable data which is comparable to or better than commercially available whole-plant systems or other custom made devices.

### Statistical analysis of the measurements

To evaluate the effect of external variables on the photosynthetic measurements of the EGES-1, statistical tests were performed. Gas exchange data of more than 70 wild-type plants, grown and measured independently over several months, were analyzed. For the measured plants, four variables were considered. Firstly, plant age, which varied between 25 and 36 days. Secondly, projected leaf area, which ranged from 180 to 1500 mm^2^. Third, the chamber in which the plant was measured. Fourth, the relative humidity (RH). As the system does not include an automatic feedback regulation for the RH, the water content of the air within the chamber varied between 55 % (that of the incoming air) and 95 %. Correlation analysis and one-way analysis of variance (ANOVA) were applied to check if any of these variables have an influence on net carbon assimilation rate or transpiration rate.

**Fig. 3:**
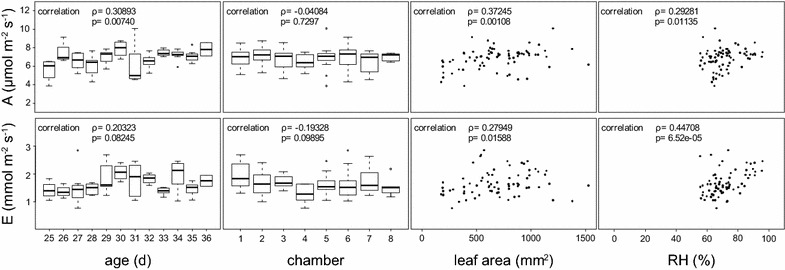
Correlation analysis of photosynthetic carbon assimilation rate and transpiration rate with external variables. *Box plot* of A (*upper row*) and E (*lower row*) against plant age, chamber in which the plant was measured, leaf area and relative humidity. In the *left upper corner* the correlation coefficient (ρ) and significance of the correlation (p) between the two variables is highlighted. *A* photosynthetic carbon assimilation rate, *E* transpiration rate, *RH* relative humidity of the incoming air

The results of this test series are depicted in Fig. 3, where all variables are plotted against A and E. Additionally, each plot shows the correlation coefficient calculated after Spearman (ρ value) and the corresponding *p* value. None of the tested variables shows a strong correlation (ρ > 0.7) to either A or E. The lowest correlation could be found between the chamber and A or E. This shows that the chamber in which the plant was measured did not influence the measurements. Weak positive correlations (0.2 < ρ < 0.4) were observed between the age and A or E as well as between leaf area and A or E. This indicates either a genuine rise in gas exchange as the plants age and grow. Alternatively, it could reflect that projected leaf area underestimates total leaf area as new leaves start to shade older ones. Shaded leaf area was shown to be ~5 % of the total leaf area for plants grown in similar conditions [2, 9], which would not be expected to have a strong influence. However, neither of the correlations were strong. More data would be necessary to substantiate them and reveal their cause. The change in relative humidity (RH) caused by transpiration is a common problem in gas-exchange measurements. Ideally, flow rates would be continuously adapted so that the RH of the incoming and outgoing air is comparable between measurements. As this is not practical for a whole-plant measuring system, the RH of the incoming air was kept stable and the RH of the outgoing air was allowed to vary. In this case, a high transpiration caused a high RH of the outgoing air. It was therefore not surprising that the highest correlation (ρ = 0.45) was observed between these two parameters. However, a high RH of the outgoing air correlated only weakly with A (ρ = 0.29). Overall, these tests show the method to be robust. Nevertheless, the data recommends measuring plants in a comparable developmental stage and leaf area, rather than plants of the same age. In this way, differences in shaded leaf area and relative humidity are kept to a minimum.

### Long term measurements performed with the EGES-1

An important advantage of our multiple chamber system is the possibility to conduct measurements for consecutive days without major user intervention. To illustrate this, eight Arabidopsis plants were measured continuously in real-time for 6 days (Additional file 1: Figure S6). At the start of each day, plants were imaged to determine the projected leaf area, which was used to normalize the data for the preceding 24 h. Leaf area increased by approximately 12–15 % each day, comparable to the growth rate normally observed in our growth cabinets. The mean A per day was stable at 6.9 µmol m^−2^ s^−1^, varying only between 6.7 and 7.1 µmol m^−2^ s^−1^ over the 6 days of measurements. Stable results were obtained also for respiration (mean = -0.9 µmol m^−2^ s^−1^, varying between −0.7 and −1 µmol m^−2^ s^−1^). E was less stable over the measurement period and decreased over the light periods from an average of 2.1–1.6 mmol m^−2^ s^−1^. In the dark, transpiration fluctuated in the first days, but stabilized later at around 0.8 mmol m^−2^ s^−1^. The changes in transpiration rate might result from a rise in relative humidity due to the increase of leaf area during the measurement period. However, these small changes in relative humidity do not affect net carbon assimilation rate.

Interestingly, after initial assembly of the chambers we frequently noted a drop in the enclosed plant’s relative growth rate for 1–2 days (judged by changes in projected leaf area; not shown), after which normal growth resumed. Therefore, a second set of eight chambers was constructed to be used in rotation. This allowed the one chamber set to be assembled around the plants in advance (without the lid) while other plants were being measured by the EGES-1 using the second chamber set.

### Measuring photosynthetic rate in different environments

With the EGES-1, plants can be monitored under different environmental conditions. To demonstrate the sensitivity of the system, Arabidopsis wild-type plants were measured under different light intensities (60, 160 and 540 µmol quanta m^−2^ s^−1^). All plants were grown under standard conditions for 3 weeks, before they were transferred to the respective light conditions. After 3 days of adaptation, two sets with eight plants each were measured for each light intensity over a 12-h light period (one set is shown in Additional file 1: Figure S7). Both A and E differed with the light intensity, as expected. At 540 µmol quanta m^−2^ s^−1^, both data sets showed an average net carbon assimilation rate of 10.6 µmol m^−2^ s^−1^, with standard deviations of 1.0 and 1.3, respectively. Likewise, the data for 160 µmol quanta m^−2^ s^−1^ (7.1 ± 0.6 and 6.2 ± 0.8 µmol m^−2^ s^−1^) and 60 µmol quanta m^−2^ s^−1^ (2.9 ± 0.5 and 2.6 ± 0.7 µmol m^−2^ s^−1^) gave highly reproducible results. The variation obtained for transpiration rate was slightly higher. For 540 µmol quanta m^−2^ s^−1^ the averages for transpiration rate were 2.3 and 2.9 µmol m^−2^ s^−1^ with standard deviations of 0.2 and 0.3, respectively. Again, reproducible results were gained for 160 µmol quanta m^−2^ s^−1^ (1.6 ± 0.2 and 1.9 ± 0.2 µmol m^−2^ s^−1^) and for 60 µmol quanta m^−2^ s^−1^ (1.0 ± 0.2 and 0.6 ± 0.1 µmol m^−2^ s^−1^). These data show that net carbon assimilation and transpiration rates can be readily monitored between batches of similarly-grown plants.

**Fig. 4:**
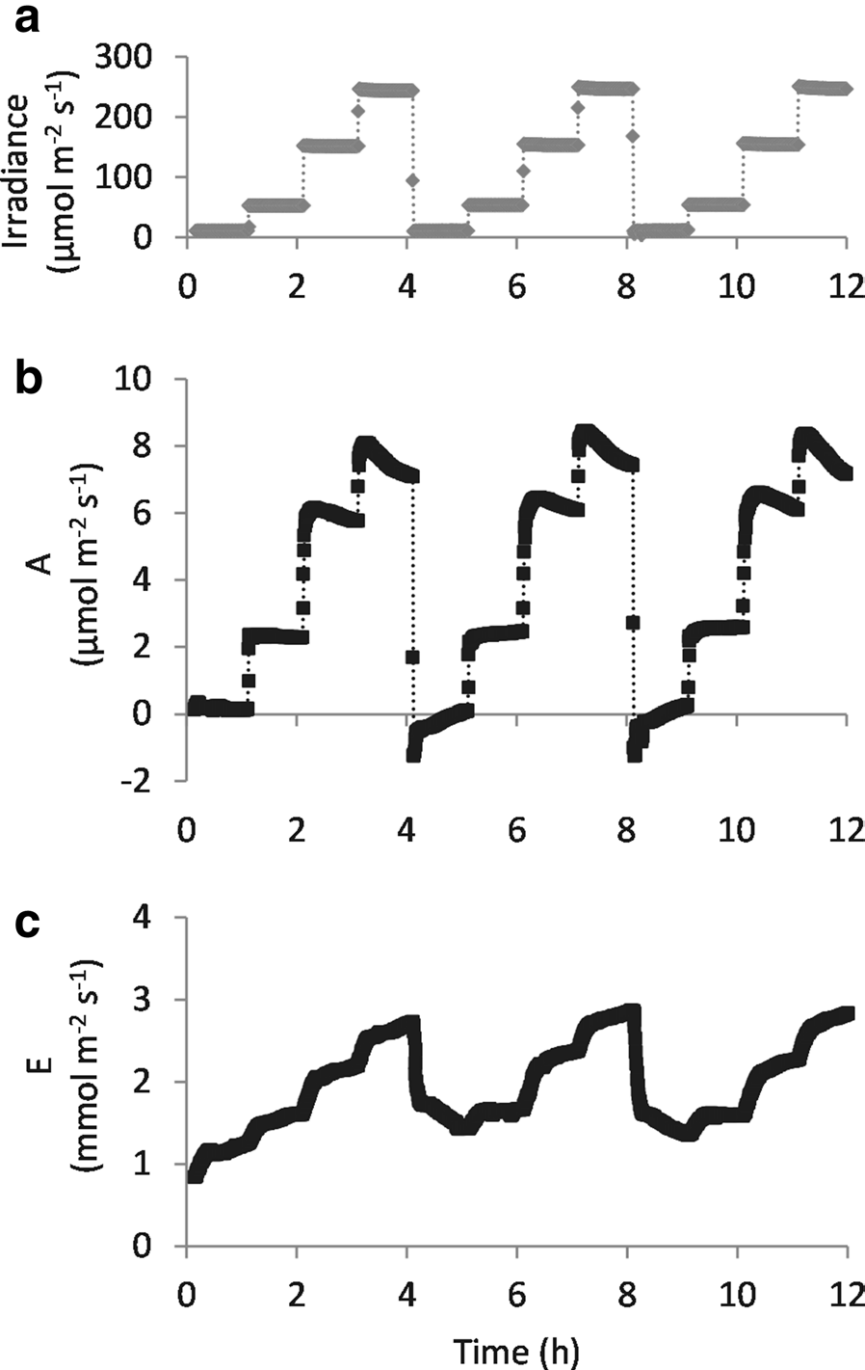
Gas exchange in changing light conditions. Gas exchange of Col-0 plants was measured in an LED chamber with light conditions changing every hour from 10 to 50, 150 and 250 µmol quanta m^−2^ s^−1^ (**a**). This cycle was repeated three times. The curves for photosynthetic carbon assimilation rate (**b**) and transpiration rate (**c**) of one example plant are depicted

Photosynthetic responses can be also monitored over shorter time scales. Inevitably, the faster the rate of change in conditions, the more limited the number of plants that can be measured in parallel. Using an LED chamber, light intensity was changed every hour and the effect on one plant at a time was measured. The measurements started with the lowest light intensity of 10 µmol quanta m^−2^ s^−1^ which was increased stepwise to 50, 150 and 250 µmol quanta m^−2^ s^−1^ (Fig. 4). This cycle was repeated 3 times. Net carbon assimilation rate reacted immediately on changes of light intensity and increased stepwise from 0.6 to 2.8, 6.6 and 8.4 µmol m^−2^ s^−1^. These results are stable over all three cycles. The transpiration rate reacted more slowly than the carbon assimilation rate. However, distinct differences in transpiration were visible for the different light intensities. On average, transpiration rate increased from 1.1 to 1.5, 2.1 and 2.6 mmol m^−2^ s^−1^. In the second and third cycles, the transpiration rates were slightly higher, especially for the lower light intensities. While transpiration rate increased during the first cycle with 10 µmol m^−2^ s^−1^ light, it steadily decreased in the second cycle after switching back from high light to the lower light intensity. It seems that 1 h of low light was insufficient for the plant to adjust stomatal conductance and to achieve a stable rate of transpiration. These data show that the effect of fast changes in environmental conditions can be followed precisely via this gas exchange system.

### Measuring net carbon assimilation rate after experimental manipulations and under stress conditions

**Fig. 5:**
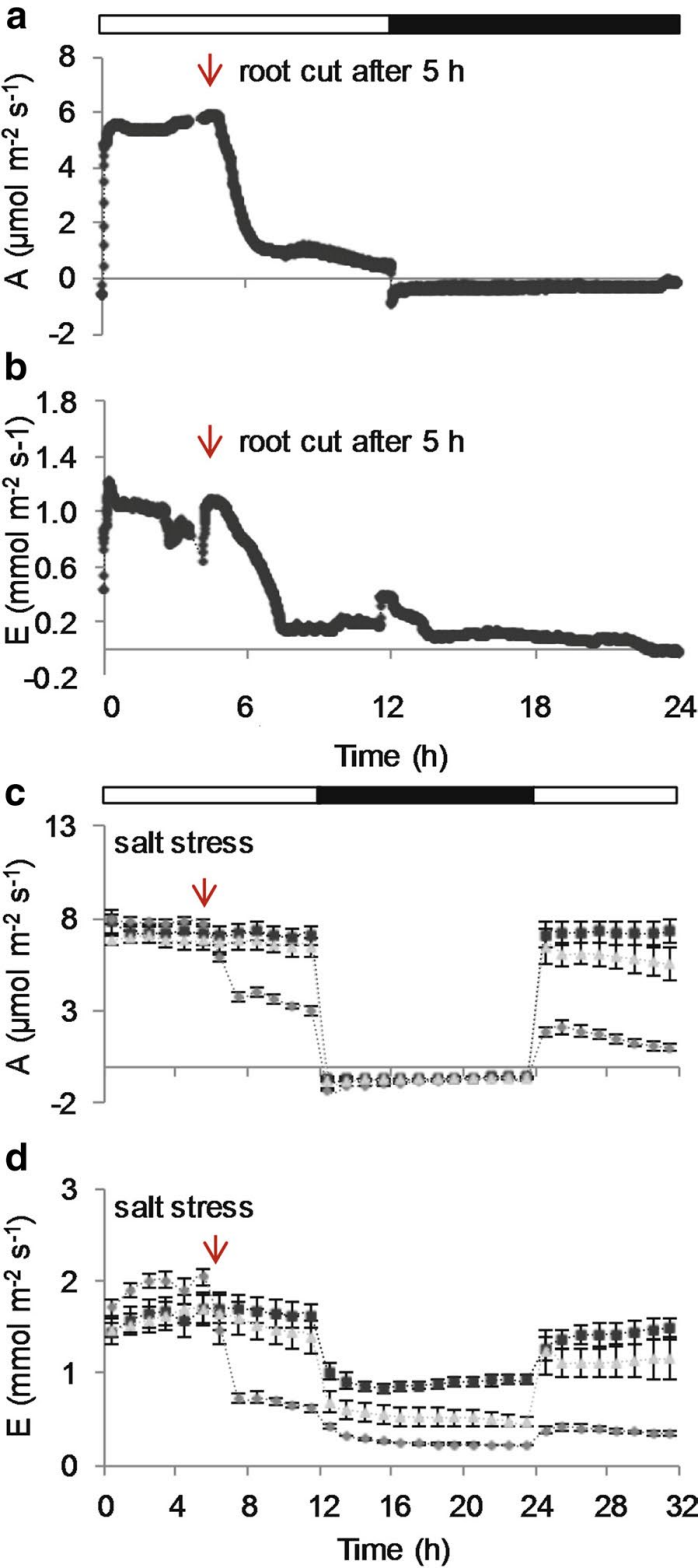
Gas exchange after mechanical damage and under stress conditions. **a** Photosynthetic carbon assimilation rate and **b** transpiration rate of a Col-0 plant from which the roots were cut 5 h into the light period. The measurements were repeated for three plants; the data for one representative plant are shown. **c** Photosynthetic rate and **d** transpiration rate of Col-0 plants under salt stress. All plants were measured for 6 h in the light period before treatment with 50 mM NaCl (*pale gray triangles*), 200 mM NaCl (*dark gray diamonds*), or left untreated (*black squares*). *Open bars* indicate the light period, *solid bars* the dark period. Mean ± SE (n = 4)

Two experiments were performed to demonstrate the effect of mechanical damage and imposed stress conditions on the plants’ net carbon assimilation rate. One led to an immediate response, while the other had a mild or latent effect on photosynthetic carbon assimilation. Both experiments were carried out using hydroponically-grown plants. To elicit a strong, rapid response from the plant, the entire root was detached from the shoot immediately below the foam gasket (Fig. 5a, b). The initial value for A was 5.84 µmol m^−2^ s^−1^. This started to decline immediately after removing the root. Within 15 min it was reduced by 10 %, and after 50 min by 50 % of its original value. By the end of the light period, the value for A had decreased to 0.5 µmol m^−2^ s^−1^. In the following dark period, respiration rate was constant but only one-third of the rate observed during the preceding night (not shown). Transpiration declined quickly after removal of the root. From an initial transpiration rate of 1.07 mmol m^−2^ s^−1^, E decreased steadily by 90 % within 3 h after cutting.

Next, salt stress was applied to a set of replicate plants and the effects on photosynthesis were observed over a longer time period (Fig. 5c, d). Initially, plants were grown in half-strength Cramer solution and measured with the EGES-1. In the middle of the first light period, NaCl was added to the nutrient solution of the plants to a final concentration of either 50 or 200 mM. Plants stressed with 200 mM NaCl solution responded immediately with reductions in both A and E, relative to untreated plants. Within the first 2 h, A declined by 50 % followed by a more moderate decrease of another 10 % until the end of the light period. Respiration rate during the subsequent dark period was slightly increased relative to non-stressed control plants at the beginning of the night, but similar to the controls later during the dark. Photosynthetic carbon assimilation rate did not recover during the next light period; it reached a peak value of 34 % compared to the controls and decreased again during the course of the day. Transpiration rate was reduced by 65 % within the first 2 h after the salt stress was applied and also declined further until the end of the light period. Transpiration remained lower than the control plants in the subsequent dark period.

Treatment with 50 mM NaCl did not have an instant effect on A, although E declined steadily until the end of the light period, finishing 20 % lower than when the stress was first applied. During the subsequent night, E was half that of the control plants. Although 50 mM NaCl did not affect A in the remaining 6 h of light, nor respiration rate during the subsequent dark, during the following light period A decreased gradually whereas in the control plants it was stable. This trend was visible for every treated plant, even though the standard error in the group was quite high. These data show that it is possible with the EGES-1 to differentiate between factors affecting A and/or E and that a reduction in E does not necessarily lead to an immediate reduction on A as well.

### Measuring photosynthetic rate in different plants

Using the different lid types, net carbon assimilation rates were measured in other plant species (Additional file 1: Figure S8), including *Nicotiana sylvestris*, *Mesembryathemum crystallinum*, (using type-2 lids) *Pisum sativum* and *Lotus japonicas* (using type-3 lids). For upright species, projected leaf area is either more difficult to determine or not a useful measure. Therefore, at the end of the measurement, plants were harvested, weighed, and the total leaf area determined to allow the expression of A on a leaf area basis. In a second experiment with an independent batch of plants, only the fresh weight was determined and the weight-to-area conversion factor from the first experiment used. Both experiments yielded similar results for each species. *L. japonicas* gave the highest values for A (~7.5 µmol m^−2^ s^−1^), while *P. sativum* gave the lowest values (~3.75 µmol m^−2^ s^−1^). We tested whether the larger chamber volume associated with type-3 lids affected measurements of A due (e.g. to slower air circulation) by varying the air flow rate (100, 120, 140, 160, 180 and 200 µmol s^−1^). The values for A were not different.

### Setup and system limitations

During our testing and use of the EGES-1, plant size and the photosynthetic capacity of the plants to be measured were the most important parameters for setting up the system. The upper size limitation is imposed by the respective lid design, and the need for an even flow of air through the chamber. Excessively large plants can obstruct an even air flow and, because of a high transpiration rate, lead to condensation that confounds reliable measurements. Theoretically, there is no lower size limitation, as long as the plants can be introduced into the air-tight chamber. However, for smaller plants, the ΔCO_2_ value can become too small to obtain reliable data. In this case, the air flow can be decreased, which leads to an increase in ΔCO_2_ and simultaneously to an increase in ΔH_2_O. However, a smaller flow rate slows the response time of the system and reduces the numbers of measurements that can be taken per measurement period. It also increases the likelihood of diffusional and/or bulk flow leaks of CO_2_ between the external air and the chamber. Therefore, the choice of air flow through the system has to be optimized in a way so that ΔCO_2_ is maximal, that ΔH_2_O is within an acceptable range, that the system has a reasonable response time, and that risk or significance of leaks is properly evaluated.

Another limitation of our system is its reliance on airflow to provide adequate air movement and mixing within the chamber. For small chambers, such as those used for Arabidopsis, the relatively high flow rate we used is equivalent to one chamber volume every 8.5 s. Nevertheless, it is still quite likely that there are differences in the micro-environments from one part of the plant to the next. For example, the thickness of the boundary layer of still, or slow moving air immediately adjacent to the leaf surface, may differ from the top to the bottom of an Arabidopsis leaf, given its flat rosette form. Similarly, in more upright species, the boundary layer might differ from one leaf to another, depending on their orientation. Such issues can be overcome in single-leaf cuvettes, where conditions adjacent to the leaf can be more precisely controlled. However, this is more difficult to address with whole-plant systems such as ours. That said, differences in micro-environments are physiologically realistic for many plants, including Arabidopsis. Consequently, neither single-leaf nor whole-plant measurements are perfect. They should be seen as yielding complementary information.

In experiments with the largest of our chambers, where the airflow rate was equivalent to one chamber volume every 44 s, no differences in net carbon assimilation was detected upon reducing the flow rate by half. This suggests that, over this range, the boundary layer resistance was not significantly different, even if our data do not establish exactly what the impact of the boundary layer on net carbon assimilation was. To investigate such aspects further, or to employ larger chambers with our system, additional steps would needed such as the use of higher airflow rates and/or the introduction of a variable-speed fan within each chamber to circulate the air.

## Conclusions

The EGES-1 is a reliable and versatile multiple chamber system to perform gas exchange measurements of up to eight whole plant shoots simultaneously and long-term in a variety of growth facilities. We have shown that it can be adapted to a range of plants by adjusting the lid design and used with plants grown on both soil and in hydroponic culture. The capacity for parallel measurements is, in our view a major advantage that significantly increased the robustness of the data obtained. Furthermore, the incorporation of independent mass flow controllers for each chamber means that the conditions for each plant are constant, regardless of whether they are being measured or not: This is an advantage as it ensures that the effects of plant handling are minimal, compared with single-leaf or single-plant systems, and it negates or minimizes issues such as gas adsorption onto chamber surfaces, as such processes would be at or close to steady state.

Our test experiments show that the EGES-1 gives accurate, stable and reproducible measurements, allowing measurements of gas exchange over timescales ranging from minutes to days in response to sudden or gradual environmental changes (e.g. light quantity, mild salt stress). Other environmental and stress parameters and their effect on photosynthetic carbon assimilation and transpiration can be analyzed with this system, both in wild-type plants and in the large number of gene knock-out or overexpression lines available today. As one recent example, the EGES-1 was used to demonstrate the inability of mutants deficient in chloroplast-localized kinases to acclimate to high light stress [14].

## Methods

### Growth conditions on soil

*A. thaliana* (ecotype Col-0), *L. japonicus*, *M. crystallinum*, *N. sylvestris*, and *P. sativum* seeds were sown on Einheitserde Typ ED 73 (Einheitserde- und Humuswerke Gebr. Patzer GmbH & Co. KG, Sinntal, Germany). Arabidopsis seeds were stratified for 3 days at 4 °C before they were transferred to a growth cabinet. All the plant species were grown with a 12-h photoperiod with the light intensity set to 150 µmol quanta m^−2^ s^−1^, and a constant temperature of 20 °C and a relative humidity of 65 %. After seedling establishment, individual plants transplanted into a new pot. Forming the soil into a slight dome facilitated the subsequent introduction of the plants into the EGES-1 stabilizing units.

### Arabidopsis growth in hydroponic culture

One-milliliter tubes filled with 0.65 % Plant agar (Duchefa, The Netherlands) were used to support plants grown in a hydroponic solution. The bottom of the tubes was cut off and the tubes were placed in a rack with the cut ends immersed in full Cramer solution [15]. To avoid exposing the solution to light, the bottom of the rack was covered with aluminium foil. After stratification for 3 days at 4 °C the seeds were transferred to a growth cabinet set to the conditions specified above. After 2 weeks, the nutrient medium was changed to half-strength Cramer solution. Thereafter, the nutrient solution was exchanged on a weekly basis.

### Gas exchange chambers

The chambers to measure gas exchange of whole plant shoots were designed and built in house (Fig. 1). Each of the chambers comprises two stabilizing units made of aluminum, corrosion resistant steel and unplasticized PVC, which can be assembled by two long bolts. A Plexiglas lid can be screwed on top to create a gas-tight chamber containing only the shoot of the plant. A rubber lip (nitrile butadiene rubber) seals the gap between the two stabilizing units. Foam gaskets (ethylene-propylene-diene rubber) seal the connection around the plant hypocotyl/stem and between the stabilizing units and the Plexiglass lid. Every chamber is connected via an inlet and an outlet to the control unit and IRGA. Sensors placed into one of the chambers to measure air temperature, showed it was similar to ambient, and that light intensity was attenuated only slightly by the Plexiglas lid.

### The gas exchange measuring unit (EGES-1)

The custom-designed system, EGES-1 (DMP Ltd, Switzerland), was built to measure gas exchange. It comprises a control unit, an IRGA (LI-7000, LI-COR Inc. USA) and a Linux computer for data acquisition (Fig. 2; and Additional file 1: Figure S1).

The control unit (Phoenix Mecano AG, Stein am Rhein, Switzerland) includes all the electrical devices; an interface USB-6008 (National Instruments, Ennetbaden, Switzerland), a converter TCC-100 (Moxa, Unterschleissheim, Germany) and eight mass-flow controllers (red-y smart Serie, Vögtlin Instruments, Aesch, Switzerland). The sensors to measure light intensity and temperature within the gas exchange chambers are connected to the control unit. PTFE tubes (Maagtechnic, Switzerland) are used to connect the control unit, the chambers and the IRGA. The tubing is attached via one-touch fittings (SMC Pneumatik AG, Switzerland).

All data from the IRGA as well as from the additional sensors is transferred to a Linux computer. A LabView application (National Instruments, Ennetbaden, Switzerland) was programmed to collect the data and to operate the control unit (The LabView source is available upon request).

### Setup of gas exchange measurements

The gas exchange measurements were performed, unless otherwise stated, with a flow of 200 µmol s^−1^ within a growth cabinet set to an air temperature of 20 °C and a light intensity of 130–150 µmol quanta m^−2^ s^−1^. The incoming air was adjusted to a CO_2_ concentration of 380–400 ppm and a relative humidity of 55–65 %. Unless otherwise specified, the outgoing air of each chamber was measured for 360 s before the system switched to the next chamber. A dead time of 90 s was introduced after each switch to allow stabilization of the measurements. After the dead time, measurements were made every 2.5 s; every 30 s the average value was calculated and stored by the LabView application.

### Determination of leaf area

Unless otherwise stated, the projected leaf area was used for determination of the photosynthetic carbon assimilation rate and the transpiration rate. For this purpose, a digital picture of the plants was taken once per day. The pictures were converted to black-and-white images and the leaf area was calculated with ImageJ (ImageJ 1.42q, National Institutes of Health, USA).

### Gas exchange data analysis

Prior to data analysis, parameters including relative humidity, light intensity and CO_2_ content of the incoming air were confirmed as stable and within the ranges specified above. The values for ΔCO_2_, ΔH_2_O, flow rate and the projected leaf area were used to calculate photosynthetic parameters according to von Caemmerer and Farquhar [16].

### Statistical analysis

For simple statistical analysis Microsoft Excel functions were used. For the correlation analysis, the variance analysis and plotting of the corresponding figures the software package R was used (R Development Core Team, http://www.r-project.org/).

## Additional file

10.1186/s13007-015-0089-z Supplemental figures S1–S8.

## Abbreviations

A: photosynthetic rate
E: transpiration rate
IRGA: infrared gas analyzer
RH: relative humidity
