# Supplementary material for: A whole-plant chamber system for parallel gas exchange measurements of Arabidopsis and other herbaceous species
Source: Plant Methods. 2015 Oct 16;11:48. doi: 10.1186/s13007-015-0089-z (PMC4609071; doi:10.1186/s13007-015-0089-z)
Supplement: Supplementary file 1 — 10.1186/s13007-015-0089-z Supplemental figures S1–S8. [file 13007_2015_89_MOESM1_ESM.pdf]

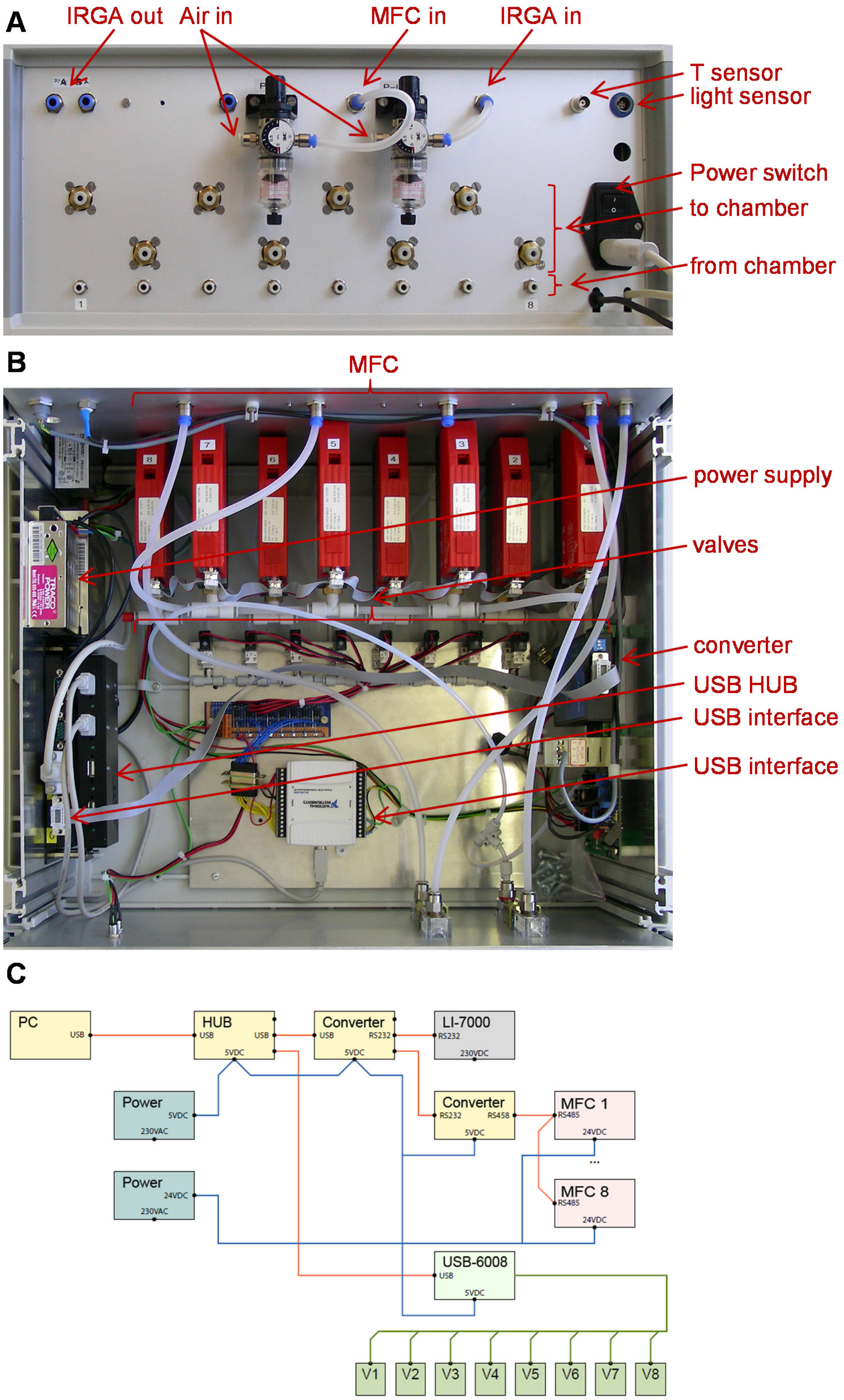

Supplemental Figure 1. The EGES-1 control unit.
Back view (A) and top view (B) of the control unit. (C) Interface topology.

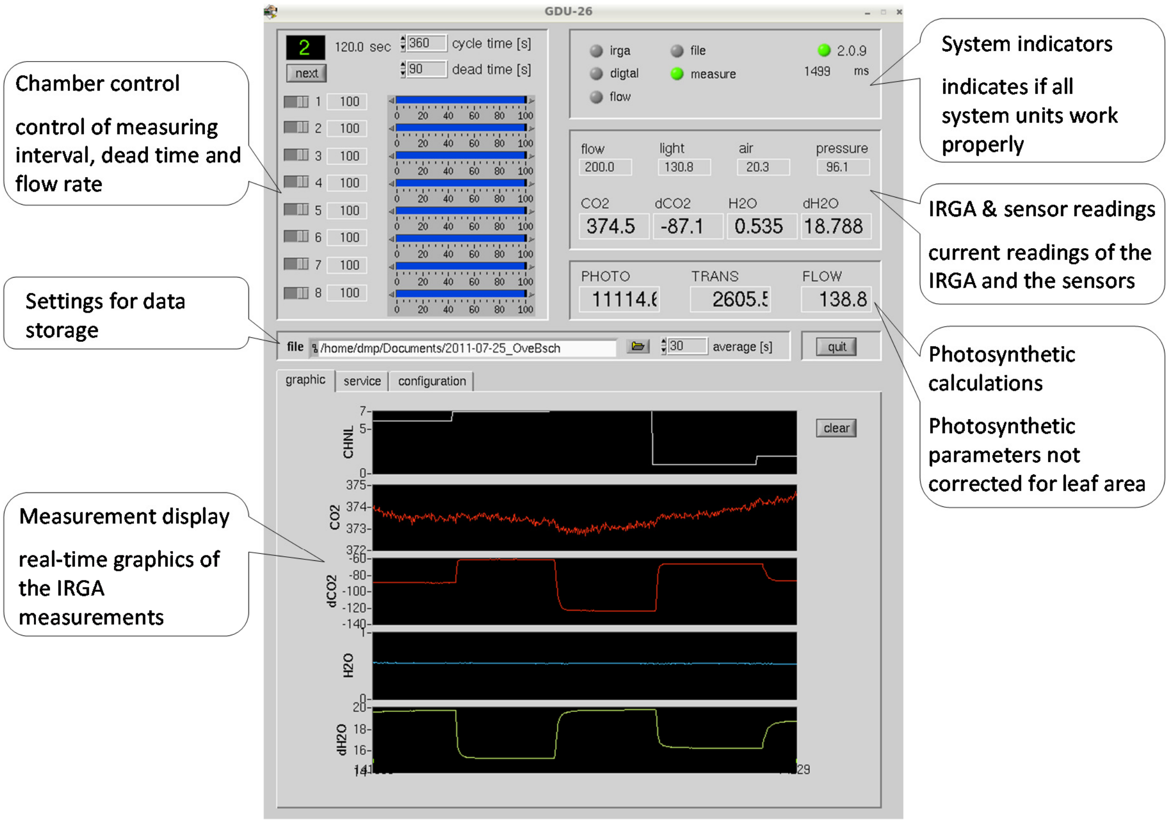

Supplemental Figure 2. LabView application to control the EGES-1 gas exchange unit.
With the program it is possible to control the gas flow, monitor the gas exchange and environmental parameters in real time and set the parameters for data acquisition.

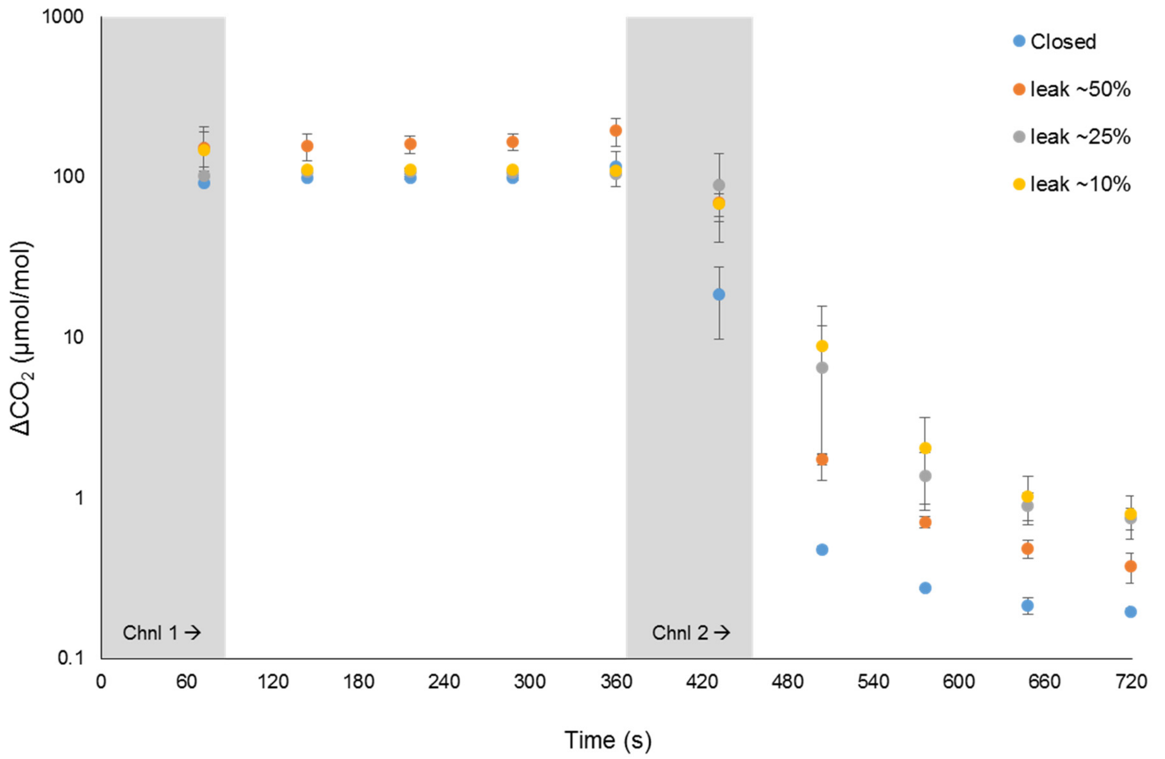

Supplemental Figure 3. Evaluation of the impact of diffusional and bulk-flow leaks.

Test experiments performed to measure CO_2 diffusional leaks using a closed chamber supplied with air containing 380-400 ppm CO_2. Ambient air measured in Channel 1 (Chnl 1) had a CO_2 concentration 100-150 ppm higher than the supplied air. Switching to the supplied air after passage through the closed chamber (Chnl 2) gave a CO_2 reading of below 0.2 ppm, indicating negligible diffusional leakage of CO_2 into the chamber during measurements (note the log scale). Comparable experiments conducted on chambers into which bulk airflow leaks were deliberately introduced (dropping the returning air flow to approximately 50, 25, or 10% of that supplied) showed that progressively larger leaks in the system influenced the time taken to reach an accurate measurement, but had minimal impact on the final CO2 concentration measured, which in all cases was well below 1 ppm. In normal experiments, return airflows were generally close to 100% and always above 80%.

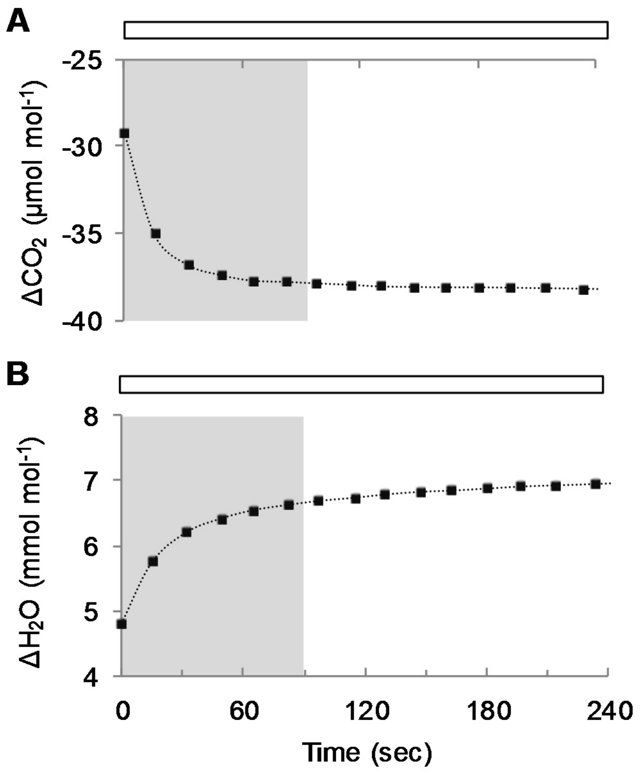

Supplemental Figure 4. Test runs of the gas exchange system.
Measurements (A) ΔCO_2 and (B) ΔH_2O values without a dead time before data acquisition. The gray shaded area indicates the data that is not stored if a dead time of 90 seconds (sec) is applied. Measurements were repeated 8 times and representative data of one plant is shown.

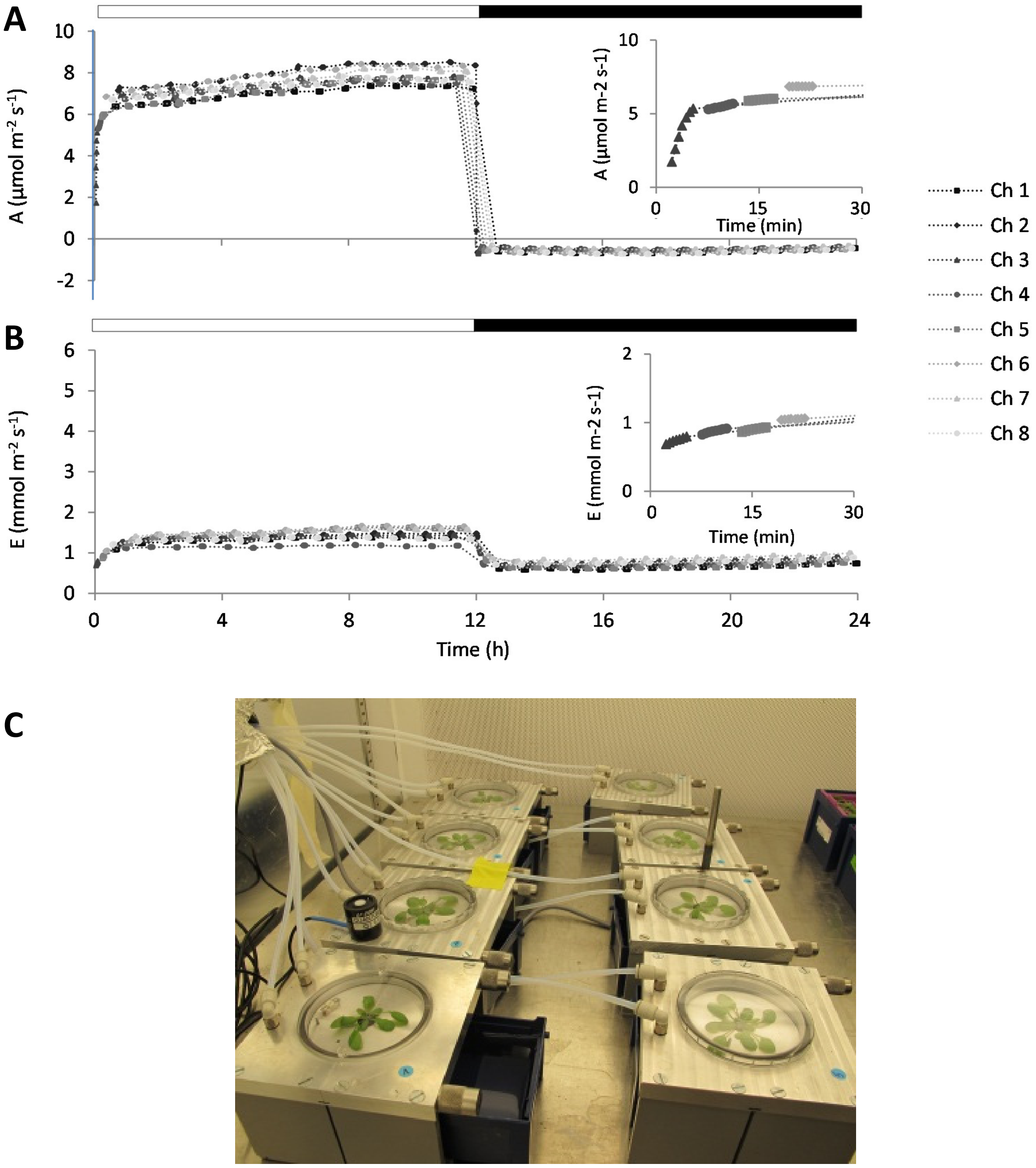

Supplemental Figure 5. Comparison of gas exchange measurements recorded in parallel with the EGES-1.

The measurements for photosynthetic rate (A) and transpiration rate (B) of each of the eight chambers (Ch 1 to Ch 8) are shown over a complete one day-night cycle (12 h light/ 12 h dark). The insets illustrate photosynthetic rate and transpiration rate in the first 30 min after the light was switched on. Open bars indicate the light period, solid bars the dark period. (C) A typical set of eight plants.

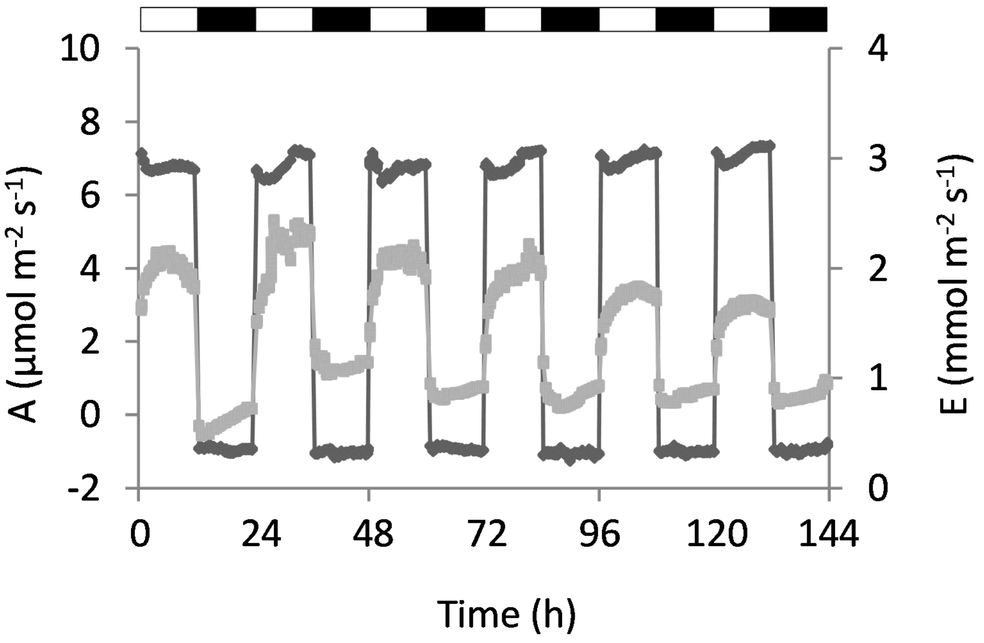

Supplemental Figure 6. Long-term measurement with the EGES-1.

Gas exchange of Col-0 plants measured continuously over a period of 6 days. A measurement of one example plant is depicted. Open bars indicate the light period, solid bars the dark period. Net carbon assimilation (A) is the dark gray line and transpiration (E) is the light gray line.

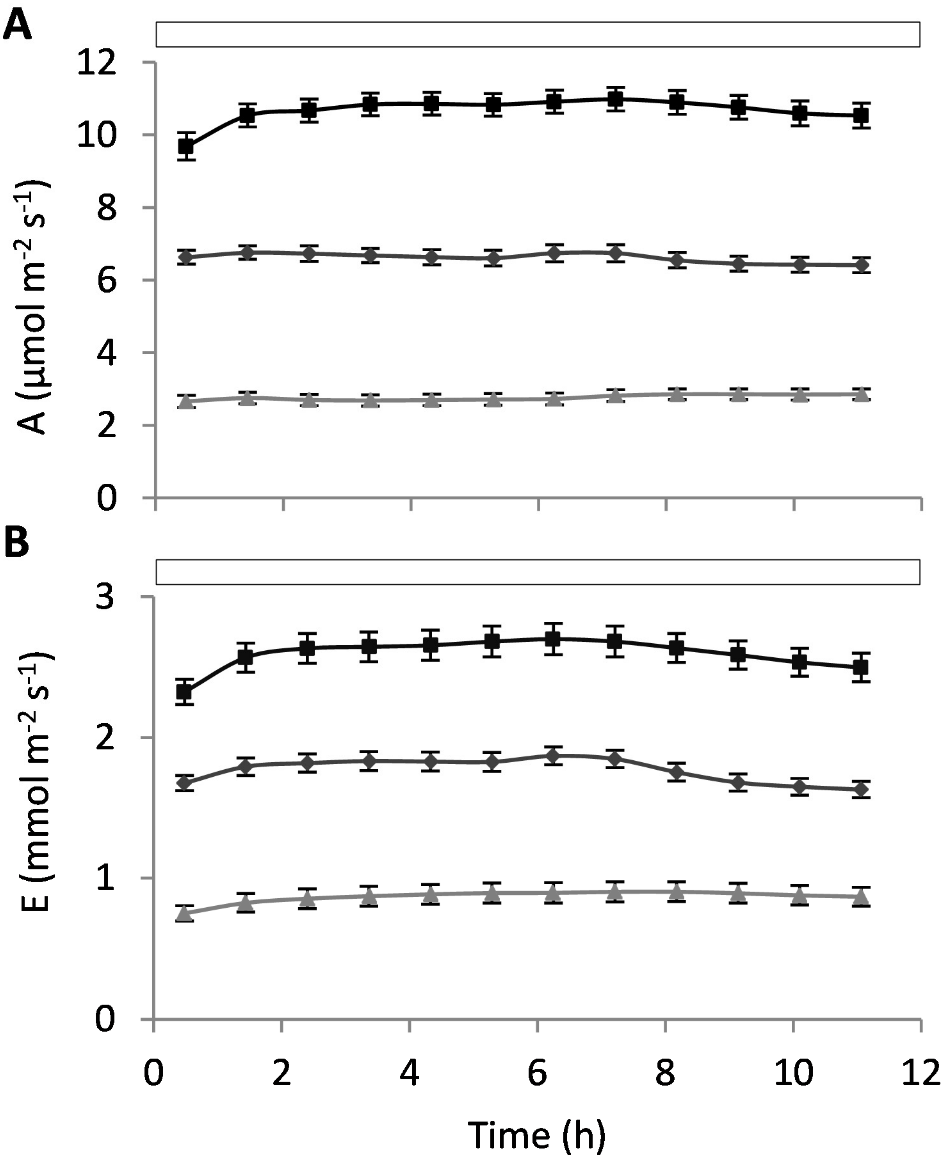

Supplemental Figure 7. Gas exchange in different light conditions.

Photosynthetic rate (A) and transpiration rate (B) of Col-0 plants in different light intensities of 60, 160 and 540 µmol quanta m^{-2} s^{-1}. Open bars indicate the light period. Mean ± SE (n=8).

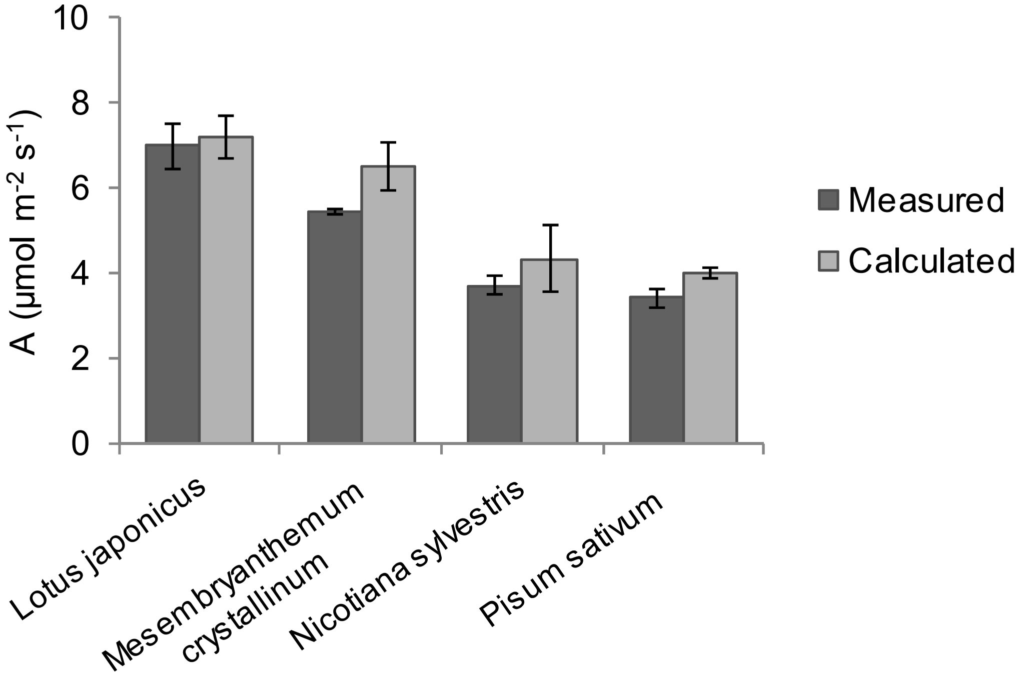

Supplemental Figure 8. Photosynthetic rate of diverse species using the EGES-1 and the appropriate lid-type.

A was measured for *Lotus japonicus*, *Mesembryanthemum crystallinum*, *Nicotiana sylvestris*, and *Pisum sativum* over the course of a day in two independent experiments. In the first experiment (dark gray bars), assimilation rates were normalized to both the leaf area and fresh mass, both of which were measured at the end of the experiment. The second experiment (light gray bars) was normalized to mass only due to the time consuming nature of acquiring leaf area on these diverse species. The data presented here is calculated using a mass-area conversion factor derived from the first experiment. Mean ± SE (n=4).
